# Immune Thrombocytopenic Purpura and Its Rare Association With a Brucella Infection: A Case Report

**DOI:** 10.7759/cureus.30049

**Published:** 2022-10-07

**Authors:** Zemin Qiu, Fang Yang, Sui Zhang

**Affiliations:** 1 Pediatrics, The First Affiliated Hospital of Jinan University, Guangzhou, CHN

**Keywords:** pediatrics, prednisolone, anti-brucella treatment, kid, brucellosis, itp

## Abstract

Although several factors such as viral and bacterial pathogens, and drugs have been widely reported to be associated with immune thrombocytopenic purpura (ITP), brucellosis is an unusual cause of this disorder. Here, we describe a patient with brucellosis with fever and purpura as the first manifestation of her illness due to immune-mediated severe thrombocytopenia. In this case, ITP responded well to the anti-Brucella treatment with platelet recovery within three days.

## Introduction

Brucellosis is one of the major public health issues in China today. It is one of the most prevalent human-animal bacterial infections of worldwide distribution. A human can easily contract brucellosis through direct/indirect contact with infected animals (goats, cattle, camels, etc.), consuming raw meat and dairy products, or inhalation of infected aerosolized particles; thus it is an occupational disease for shepherds, abattoir workers, and veterinarians [1]. It is a multisystem disease containing a broad spectrum of clinical manifestations such as fever, rash, weakness, fatigue, night sweats, and arthralgias [2]. But thrombocytopenia during the clinical course is not common, with an incidence varying from 1% to 8% in adults [3].

## Case presentation

A two-year-old girl was referred to the pediatric department with a fever and purpura. Her previous history revealed the presence of an intermittent fever, which usually occurred in the afternoon and at midnight, for over two weeks. She presented with the complaint of intermittent right wrist pain and continuous night sweats 10 days before admission. No fracture was found on the wrist X-ray. Four days prior to admission, a complete blood count (CBC) demonstrated thrombocytopenia with platelets 12 × 10^9^/L. Asymmetric pinpoint bleeding spots on both lower extremities were found three days ahead of admission. Considering the possibility of idiopathic thrombocytopenia, she was prescribed 2g/kg immunoglobulin therapy at the local hospital. Thereafter her platelet fluctuated between 58 to 68 × 10^9^/L. For further examination and treatment, she was referred to our pediatric department.

Physical findings showed her body temperature was 36.4°C, blood pressure 103/50mmHg, and heart rate 102bpm. No obvious dry and wet rales, no pathological heart sounds, or heart murmurs were heard; the abdomen was flat with no pressure pain or rebound pain. Slight conjunctival pallor and old rash on both lower extremities went along with no hepatomegaly, splenomegaly, and lymphadenopathy.

**Figure 1:**
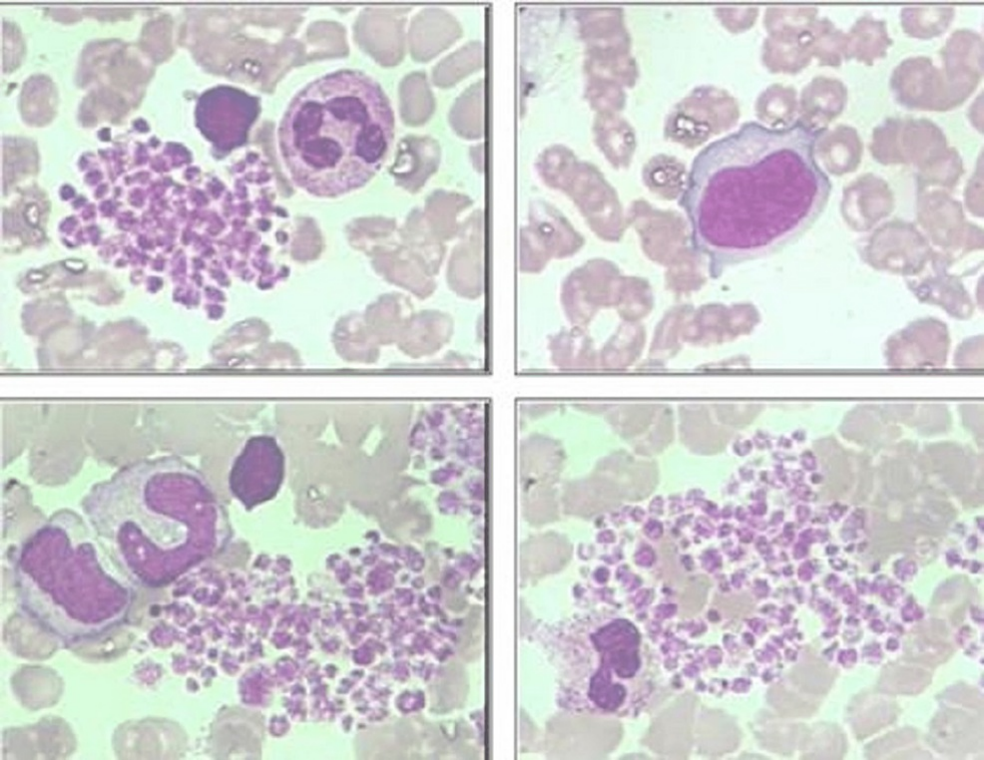
Peripheral blood smear shows normal morphology and proportion of blood cells as well as clustering distribution of platelets caused by EDTA. EDTA: Ethylene diamine tetraacetic acid

Initial CBC suggested it was hard to count the platelets. Peripheral blood smear showed clusters of platelets due to ethylene diamine tetraacetic acid (EDTA) (Figure 1). Considering the possibility of EDTA-dependent pseudothrombocytopenia (PTCP), we replaced sodium citrate with EDTA. The second CBC revealed thrombocytopenia with platelets 67 × 10^9^/L, hemoglobin 103g/L, white blood cells (WBC) 6.94 × 10^9^/L, and C-reactive protein (CRP) 3.4 mg/L. Serological tests including antinuclear antibody (ANA), rheumatoid factor (RF), and hepatitis B virus were negative with a normal level of alanine transaminase (ALT) and aspartate aminotransferase (AST). Complement component 3 (C3) level was 1526mg/L (normal range: 800-1800mg/L), with complement C4 level of 246mg/L (normal range: 100-400mg/L). Positive glycoprotein (GP) IX, GP Ib, and guanosine monophosphate (GMP) 140 were detected in the idiopathic platelet antibodies program. Therefore, the patient was diagnosed with immune thrombocytopenic purpura (ITP). We hesitated on whether to initiate the standard treatment of prednisolone due to her relentless fever.

**Figure 2:**
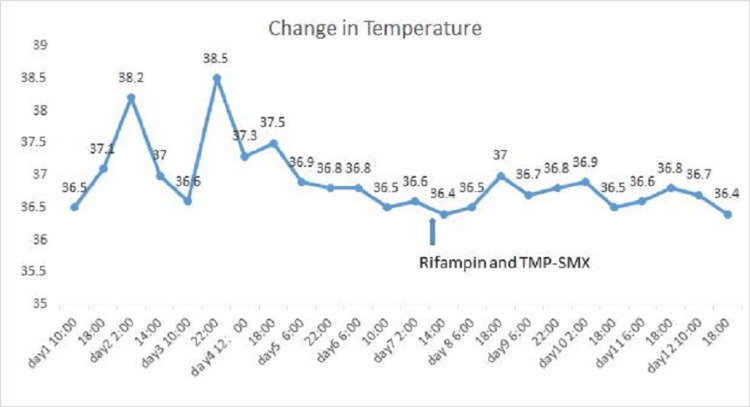
Temperature change in patient after admission and treatment TMP-SMX: Trimethoprim-sulfamethoxazole

However, our patient still had an intermittent fever when we were investigating the etiology (Figure 2). We continued to monitor the dynamic change of the blood count in case of a sudden drop in platelets, which fluctuated between 58 to 68 × 10^9^/L. On day seven of hospitalization, a blood culture at the local hospital confirmed the infection of *Brucella melitensis*. A specific agglutination test for brucellosis was documented at a titer of 1:400. We re-checked the medical history and found that the girl used to wander to the nearby slaughterhouse. Since *B. melitensis* was the source of infection in our patient, she was treated with rifampicin (12mg/kg.d) and compound sulfamethoxazole (20mg/kg.d). Three days later, her platelet returned to normal to a level of 125 × 10^9^/L.

## Discussion

According to the data of the Chinese Center for Disease Control and Prevention, the epidemic of brucellosis in China has been successfully controlled since 1978 [4]. But since 1995, there has been a resurgence, especially in the southern cities. The present case is from Guangdong, also in a non-pastoralist area in the south. This suggests that general attention should also be given to the non-infected cities in terms of prevention and control.

*Brucella melitensis* is a Gram-negative bacteria that can infect many soft tissues and organs, so brucellosis is usually misdiagnosed for its various clinical manifestations such as fever, rash, weakness, fatigue, night sweats, and arthralgias [5]. As shown in previous literature, a variety of fever patterns has been observed, such as typical undulant fever, mild but relapsing fever, or protracted fever [6]. But in our patient, the fever was not regular nor had a pattern. She had an intermittent fever, sweating, fatigue, osteoarthritis, and thrombocytopenia. It was hard to find clues to diagnose brucellosis since she had a non-specific febrile illness without localizing signs. Her previous contact with livestock, the culture of the organism detected in the blood, and Brucella total antibody titer ≥1:160 were our basis for the diagnosis. One or more sites of infection (such as osteoarticular disease, liver abscess, endocarditis, or meningitis) are the common complications of brucellosis, which occur more frequently in adults than in children [7]. Mild anemia and leukopenia are the most common haematologic disorders. Severe thrombocytopenia like in our patient is rarely seen. Except for the possible peripheral arthritis and thrombocytopenia, our patient did not have other complications. For those who have developed central nervous system brucellosis, pleocytosis (predominantly mononuclear cells), mild to moderately elevated protein levels, and hypoglycorrhachia are easily observed. In most cases, the blood culture samples are positive after seven to 12 days [8,9].

Our patient presented with severe purpura and thrombocytopenia that was initially diagnosed as ITP, which responded poorly to a sufficient dose of intravenous immune globulin (IVIG). Intermittent fever suggested potential unexplained infection. Thus we postponed the corticosteroid treatment and continued to monitor the temperature. Young et al. reported a considerable response to a short-term trial of high-dose corticosteroids for most patients with Brucella-induced ITP [10]. Our team believed the emphasis of therapy should be on clearing the infection instead of corticosteroid treatment because the so-called thrombocytopenia is mostly attributed to bone marrow suppression or hypersplenism [11]. Another intriguing phenomenon is clusters of platelets due to EDTA. Two case reports from Salama et al. described this phenomenon of ITP accompanying PTCP. A possible mechanism might be chelation of calcium ions by EDTA leads to a conformational change in the membrane proteins of platelets [12]. The exposure of antigenic determinants combined with autoantibodies activates platelet fibrinogen receptors, which in turn cause platelets to clump together. Aggregated platelets become larger and can be easily classified as a red blood cell count with a similar volume when tested in a fully automated blood cell analyzer. Regimens for the treatment of children aged more than eight years with brucellosis include oral doxycycline plus rifampin, oral doxycycline plus streptomycin, or oral doxycycline plus gentamicin [13].To avoid dental staining and nephrotoxicity, we chose both trimethoprim-sulfamethoxazole (TMP-SMX) plus rifampin rather than doxycycline and streptomycin, for six weeks.

## Conclusions

In non-pastoralist/non-infected cities, children with brucellosis are easily misdiagnosed, especially those with untypical symptoms. The diagnosis of brucellosis should be based on a combination of clinical presentation, history of exposure, and hematology tests. The treatment of Brucella-induced ITP should be focused on anti-Brucella therapy rather than the use of corticosteroids.
